# Natural History of Cinchona, (Peruvian Bark)

**Published:** 1849-12

**Authors:** 


We are indebted to the kindness of Professor Benjamin Silliman, Jr., for the following translation, on a subject of much interest to all well informed physicians.

Art. III.—*Natural History of Cinchona, (Peruvian Bark.)* By Dr. Maddell. Translated from the Journal de Pharmacie et Chemie, for Sept. 1849.

In 1843, M. Castlenau had charge of a scientific expedition into the interior provinces of Peru and Brazil. Dr. Weddell was appointed by the Museum of Paris to accompany him, charged with the special study of many important botanical questions, and of other branches of natural history. After two years of exploration in common, Dr. Weddell separated from M. Castelnau upon the confines of Mattogropo, in order to pursue his researches in a different direction, continuing them until 1848. The history of Cinchona, so controverted and so obscure, particularly fixed his attention, and the results of his laborious researches are found in the work whose title is prefixed. This beautiful work (a magnificent folio volume with numerous engravings) appears so important, in a pharmaceutical point of view, that we do not wait for its completion, to notice it. Dr. W. having kindly put the first sheets of his work in our hands we cannot but make some extracts in detail, which call forth the most lively interest in the young savan whose zeal, intelligence, and courage have accomplished so much.

Since Condamine, who was the first to make the cinchona tree known in Europe, even to the illustrious travelers Humbolt and Bonpland, to whom we owe the first observations upon the geography of this race of plants—a crowd of savans of every country have made it the object of their researches, and the list only of the published works upon this matter would fill many pages.

The origin of the cinchona bark was, for a long time, a mystery. Condamine first raised the corner of the veil, but it was not removed for a long time. Joseph de Jussieu accompanied in 1735, as botanist, the expedition sent by the Academy of Sciences to measure a degree of meridian under the Equator. He visited nearly at the same time, as the celebrated astronomer, the forests of Cinchona in Loxa, those of upper Peru, and penetrated almost even to the frontiers of Brazil. A succession of unhappy accidents hindered the publication of the results of his researches. He returned to Europe only in 1771, deprived of his reason, after an absence of 36 years.

Thirty years later, two expeditions were engaged to explore the region of cinchonas in Lower Peru and New-Grenada; one directed by the celebrated Mutis, the other by Ruiz and Pavon. The immense investigations of thos[illegible]

naturalists did not advance the history of this plant as much as they had a right to expect.

Since the observations of M. M. Humbolt and Bonpland, who subsequently surveyed the same districts, the region where the cinchona thrives, has been greatly extended by the discovery of new districts, and commerce has been enriched by a great number of species which were before unknown.

Up to the year 1775 nothing was known concerning the market of other species of cinchona than those of Loxa. It was not until 1772 that Mutis observed, for the first time, this invaluable tree in the neighborhood of Santa-Fé de Bogota—the epoch at which also Europe commenced to receive cinchona bark which had not doubled Cape Horn—but which had been embarked directly from the Atlantic ports of New Grenada. Some years later the authors of the "*Flore Péruvienne*," studied the species of Lower Peru, and of Northern Lima, and again commerce was enriched by their researches. The only species which then remained unknown in Europe, botanically speaking, were those inhabiting the vast extent of country which stretches away from the back of the grand Cordilleras to the south of these latitudes. In spite of the efforts of Joseph de Jussieu and of the botanist Thaddeus Haenke, the science of the cinchona had not preserved a single result of their voyage.—The work of M. Weddell has for its object to make known the species which he has observed in those regions during the years 1845, 1846, and 1847. The immense ravages of commercial enterprize upon the cinchonas in those parts, to the almost destruction of the ancient forests, rendered a new research on this subject quite necessary. At one period, when the consumption of these barks was increasing, it was useful to call attention to such species as at a future day might replace the *cinchona calisaya*, the exhaustion of which became more and more probable. These species, although they may be less rich in the active principle, offer still by their abundance, some security against the possibility of our being deprived of one of the most precious products of the vegetable materia medica.

M. Weddell entered Bolivia in the month of August, 1845, by the country of the "Chiquitos" Indians. The character of the soil of this province is altogether incompatible with the existence of the true cinchonas. A great part of its surface is low and level, and during the wet season is inundated. In the month of November he traveled south, reached the Rio Grande, traversed the province of the Cordillera as far as Farija, where he arrived in January, 1846. This laborious journey had for its object, to determine, with precision, the southern limit of the cinchona region. Dr. Weddell gave the name of *cinchona australis* to the species which he discovered—like an advanced sentinel—at that extreme point, near the nineteenth parallel of southern latitude. In the month of August following, he visited some of the large cities of Bolivia. At Coquimbo ("Cochamba,") began a curious phase of his journey. He traversed near the great chain of the Andes, with the design of visiting Paz, where the great trade in cinchona bark is driven. The Andes present at this point, a long and beautiful series of natural terraces, upon which the traveler gradually descends, passing successively in review every variety of climate, and all the shades of vegetation which correspond with them. The different species of cinchona were multiplied under his eyes. Almost from the commencement of his entrance into the province of Euquisies, he took occasion to study the plant which produced the cinchona calisaya, the most valuable of all the barks, by reason of the great proportion of quinine which it contains. He gave to this plant, hitherto unknown to botanists, the name of *Cinchona calisaya.* At Palca he was apprised that he would discover, upon the banks of the Rio Ayopaya, an immense forest of cinchonas, which had as yet been unapproached by any explorer. In the province of Yungas, the most rich and fertile in Bolivia, he procured the most precise information regarding the mode of procuring, of preparation, the sale and adulteration of the barks which he desired to study.

In 1847, before the rainy season, M. Weddell retraced the pass of the grand Cordillera. The city of Sorata or Esquibel, situated upon the western slope of the Andes, at the base of one of its highest peaks, is reputed as one of the chief sources of the Bolivian cinchonas, but it is in reality only a point of transit for the products of the valleys of the interior. It was towards these that he turned his steps in passing over the snows of Illangha. The Rio Tipoani, the Pactole of Bolivia, there takes its source. One of the most frightful roads in the world, leads along the ravine of the same name, and conducts the traveler to the city of Tipoani, a pestilential place, which the thirst for gain alone has rendered habitable.—As valuable as gold itself, the cinchonas are found throughout this whole region, but already the large trees begin to disappear. For the purpose of studying the points yet untrodden, M. Weddell embarked upon a raft which he had constructed, and by means of which he happily descended the rapids of the Rio Tipoani; he afterwards visited also the mountains of the Rio Tumache.

This exploration finished, he ascended with his raft the Rio Mapiri, and rejoined the paths which thread the forest toward Aten and Apolobamba, where he arrived worn out with fatigue and overcome with fever, of which he had taken the germ upon the coasts of Tipoani. There the country takes on a more pleasing aspect. The forests have disappeared, or occupy only the horizon—the eye beholds, in every direction, beautiful grass-covered hills, clumps of shrubbery, and, succeeding these, charming groves. He there discovered several species of cinchonas which exceed but little the height of shrubs, and the flowers of which load the atmosphere with their delicious perfume.

The city of Apolobamba is the centre of one of the oldest explored parts of Bolivia, and for a long time its forests have been stripped of cinchonas.

At the close of 1847, M. Weddell visited the province of Coraboya, one of the most interesting in Peru. It is divided by the Cordillera into two regions. One of these embraces a long series of valleys, which furnish to this day the greater part of the cinchona exported from the Peruvian republic. It would be difficult to give an idea of all the treasures of vegetation which are envaled in these solitudes. The thirst for gold has peopled these recesses many times, but the forest has resumed its empire, and the axe of the woodcutter alone now disturbs their silence.

Here we quote from this learned traveler, as follows: "The name of *cascarilleros* is given," says M. Weddell, "to the men who cut the cinchona in the forest and to all those who engage in this branch of trade. The woodsmen, raised to this laborious calling from infancy, and accustomed by instinct to guide themselves in the midst of dense forests, with no other compass than the tact of the child of nature, travel as surely in these inextricable labyrinths as if the horizon were open before them.

The only season improper for the collection of cinchona is that of the annual rains—which is hardly equal to our winter in length. If some pretend that the time of the ascent of sap is the most favorable for attacking the tree, their precepts are disregarded in practice, and the exception of the rainy season is made only because of the physical obstacles which accompany it. The cutters do not in general seek the cinchona on their own account—they are more commonly engaged in the employment of some merchant, or of a small company. One responsible man is sent with them to the forest, with the title of major-domo. He is charged with receiving and examining the bark which is collected from various parts of the forest, and is morover employed in the care and distribution of the provisions. The first care of those who contemplate an enterprise of this nature in regions before unexplored, is to employ experienced woodsmen, called *diestros*, or *praticos.* The duty of these is to penetrate in diverse directions into the forest, until they come to a place profitable to be worked. They ought also to state the presence and quantity of cinchona, as well as the direction in which the trees are, and to bring specimens which will enable the quality of the bark to be judged of. This raconnoisance is the most delicate part of the operation, and demands of the men employed in it a well proved patience and sagacity. It is upon their report that the chances of success are calculated. If these are favorable, it is necessary to open a path to the point which may serve as the center of operations; from that moment all that part of the forest which is contiguous to the new road, becomes provisionally the property of him who cuts the path, and no other cascarillero can work there.

The major-domo has hardly arrived with his cutters in the neighborhood of the point of labor, when he chooses a site favorable for the location of the camp—as near as possible to some spring or river. There he constructs a shed, or light house, to protect the provisions and the products of the cutting; and if he forsees that he will be obliged to remain for a long time in one place, he does not hesitate to plant maize and beans. Experience has indeed shown that one of the grand elements of success in labors of this kind is an abundance of provisions. The cascarilleros, meanwhile, scatter themselves, one by one, in the forest, or by small bands, each carrying in his poncho, and swung over his back, provisions for many days, and the covering which is to form his bed. Thus it is that these poor people have need to put in practice all the courage and patience of which their calling is so fruitful. Obliged constantly to keep in hand their axe or knife, to remove the numberless obstacles which arrest their progress, the cascarillero is exposed by the nature of the ground, to an infinity of accidents, which may often involve his very existence.

The quinquinas rarely exist alone, a single tree in a place, but they form groups more or less thick, dispersed here and there in the midst of the forest. The Peruvians gave them the name of manchas. or "spots." At other times they live completely isolated. When this is the fact the cascarillero displays all his skill to discover them. If the position is favorable, it is upon the top of the trees that he fixes his eyes. Even with much slighter indication he recognizes the object which he seeks. A slight play of colors, peculiar to the leaves of certain species, a particular color of these organs—the appearance produced by a great mass of flowers, enable him to recognize the top of a cinchona tree at a very great distance. In other circumstances, he confines himself to the inspection of the trunks, the external appearance of the bark often being remarkable. Frequently, the dry leaves, even, which he recognizes while looking on the ground, suffice to indicate to him the near approach to the object of his search.

It is interesting to watch the Indian, through the openings in the forest, darting in and out of sight, and then seeming to scent the ground, he steps like an animal pursuing its prey, throwing himself suddenly upon the form he sought for, he stops at the trunk of the tree whose presence he as it were divined. Sometimes, however, the researches of the cascarillero are not so favorable in their results. Often they return to the camp empty handed, and their provisions exhausted, having sometimes discovered, upon the flank of the mountain, indications of the tree, from which they are separated by a torrent or an abyss.

To strip the tree of its bark, they make an incision with the hatchet a little above its root, taking care to lose nothing by denuding it, except at this point, where the bark is thickest. They have a habit of digging the earth about its circumference, that the decortication may be made complete.

It is rare, even when the trunk is cut through, for the tree to fall immediately, being sustained by the wild vines which interlace it, and by the neighboring trees; obstacles which it is the duty of the cascarillero to overcome. I remember once to have cut through a large trunk of the cinchona, in hopes of obtaining the flowers, and after having cut down three neighboring trees, to have seen it upright, maintained in its position by the vines which were attached to its top, and which sustained it in the manner of the shrouds of a vessel. When, at last, the tree is down, and the branches which could prevent its fall, have been cut, the outer bark is made to cleave by being rubbed, or better, by being struck with a small wooden mallet, or the back of the axe, and the live part of the bark being exposed is cleared by the brush being then divided through its entire thickness by incisions at uniform distances. It is then separated from the trunk by means of a knife or some similar instrument—the strips being removed entire if the position of the trunk is favorable. The size and regularity of the strips necessarily depend more or less upon these circumstances, yet, in general, for the convenience of transportation and ease of prepartion, they seek to give them the length of four or five decimeters, and a width of eight or ten centimeters. The bark of the branches is separated from that of the trunk, with the exception of that which is not rubbed, custom requiring that the exterior surface or epidermis should be preserved. Sometimes in commerce, though rarely, all bark deprived of its epidermis is rejected, not that they suppose that any particular virtue is contained in it, but it furnishes distinctive characters, easily known, and at the same time difficult to counterfeit. The cascarilleros find great care necessary to preserve this upon certain species which easily decay. They have in many places the custom of cutting the tree three or four days before decorticizing, that desiccation may commence, and the different layers of bark shall adhere more closely.

The details of drying vary a little, the bark from the branches and small stems, designed to make the cinchona which is rolled " én *canuto*," or in scrolls, is exposed to the sun, and thus acquires the desired cylindrical form.—But that which comes from the larger trunks, and which forms the flat cinchona, and which they call *tabla* or *planca*, must be submitted, while drying, to a certain pressure to avoid its being warped and twisted. For this purpose, after a short exposure to the sun, they pile one slab on another, in square stacks, alternating, as boards are sometimes arranged in lumber yards, to keep them flat; and when thus arranged, they load them with some heavy body. The next day the bark is again exposed to the sun, and again pressed; and thus the process is repeated until the material is dried. Such is the usual mode of preserving Peruvian Bark, subject to such modifications as may arise from the peculiar habits of particular districts.

Sometimes, either by design, (to increase its weight) or accidentally, the bark is imperfectly dried. This is always a source of deterioration. After all this tedious labor, it still remains for the cutter to bring his spoils to the camp, which difficult task he accomplishes by transporting it on his shoulders.

It is hard to conceive the toil which he thus endures—from five to twenty days being frequently required in this task. And when we remember the trifling sum which is paid for such painful labor, it is hard to conceive that men can be found who are willing to undertake it.*

The major-domo attends to the packing of the bark.—He makes the cutters submit to a re-measurement and assortment of their lots, which, if they have a convenient place, are dried anew—the bad pieces rejected and their weight much reduced. They are then packed in a woolen sack, brought for this purpose, and these bales are then transported either on the backs of men or of animals, to the depots in the cities, where, in general, an exterior envelope of skin is put on the bundles, which in drying, acquire thus a great degree of firmness. In this form they are known under the name of surons, and in this state they arrive in Europe. Their ordinary weight is from seventy to eighty kilogrammes, (one hundred and forty to one hundred and sixty pounds) but they are often seen much lighter.

After these details, it is easy to see how incorrect have been the ordinary notions, formed by most persons, about the mode of procuring the cinchona. Some supposed that it was subjected to a special surveillance. Others have imagined that the trees were cultivated in closed parks or plantations, and treated like the cork trees of France. It behooves us well to consider the present mode of procuring this precious product, for we are at the mercy of the semi-barbarians, who now practice a method which must soon destroy, or render extremely

---

* In general, before the product reaches the coast it has changed hands three or four times, and at each change its price is advanced.—Therefore the price asked for the cinchona in Europe, bears no proportion to its cost in its native forests. At Pelechuco, for example, a kilogramme (rather over two pounds) of the first quality costs only one franc and a half, (say twenty-eight cents) and for the same quantity the Paris manufacturers pay twenty francs (nearly four dollars). In other places I have seen paid for ordinary cinchona only six, and even four piastres the quintal.

rare, the whole race of trees which produce the Peruvian Bark.* The restoration of the trees, either by suckers or second growth from the stump which has been felled, or by seeds, is almost impossible. It is true that some check might be put on the destruction of the seeds, by a suitable guard of inspectors, but an inspector of forests in France, and a forester in America, where the forests may extend over 20,000 square leagues, is quite a different office.

Two methods suggest themselves to remedy this evil—one is to limit the exportation to a quantity equal to the production of the forests; the other to cultivate lhe tree. The former would be the most safe, but it is to be feared that already the consumption has so far exceeded the production, that this mode is out of the question. It is therefore necessary to cultivate the tree, and posterity will bless the nation who, by adopting this plan, shall rescue the Peruvian bark from destruction.

[The above translation, made by Prof. Silliman, Jr., will create a desire to see M. Weddell's work. It is strange that so little has heretofore been known about the botanical history of the Cinchonæ tribe, producing, as it does, a medicine once called divine, in Europe. And this is the more singular, from the fact that intelligent minds have enjoyed ample opportunities for obtaining facts.—

---

* The Bolivian government has granted a monopoly of the Barks of Bolivia to the company of Paz, with the right to export annually 4,000 quintals of ten pounds Spanish each. Not content with this restriction, the present moment they are accused of going beyond their rights. What then, would be the fact, if all restrictions were removed, if there existed the same restrictions elsewhere, and particularly in Peru, when the exportations are raised in some years to an almost incredible amount.

In New Grenada, when the rage for procuring the bark was at its height—as at the commencement of the present century—in 1806 alone, from the single port of Carthagena, the quantity of bark shipped amounted to the enormous figure of 1,200,000 pounds. At this day, on the contrary, they export hardly as many arrobas.

Even Dr. J. J. Von Tschudi, with all his fine means, has given but a meager account. But what little he says, possesses some interest, in view of M. Weddell's fears of the extinction of the plant. Von Tschudi says: "The mountains of Herancico, which once furnished all the apothecaries of Europe with "the divine medicine," are beginning again to yield supplies. *From the roots of the trees a vigorous aftergrowth has commenced.*" * * * * "The inhabitants of the Peruvian forest drink an infusion of the green bark as a remedy against intermitting fever. I have found it in many cases much more efficacious than the dried kind, for less than half the usual dose produces, in a short time, convalescence, and the patient is secure against returning attacks."*

From the fact mentioned by this intelligent author, that "a vigorous aftergrowth," springs up from the roots, we should suppose the best method to guard against the destruction of the plants would be for the government to prohibit the cascarilleros from felling the trees near the roots, and more especially to forbid the practice, mentioned by M. Weddell, of digging around the roots."

---

Art. IV.—*Practical Medicine and Surgery.* By T. S. Bell, M. D., of Louisville, Ky.

In the November number of this Journal, I gave a programme of the state of anesthesia, with a view of calling the attention of medical men to the necessity of prudence in the use of such powerful agents as chloroform and ether. It is a source of no ordinary satisfaction to know that those remarks have been received in the spirit in

---

* Travels in Peru, by Dr. J. J. Von Tschudi.

